# Reference gene selection for transcriptional profiling in *Cryptocercus punctulatus*, an evolutionary link between Isoptera and Blattodea

**DOI:** 10.1038/s41598-020-79030-6

**Published:** 2020-12-17

**Authors:** Zhen Li, Xiangrui Li, Qingwen Zhang, Ling Yuan, Xuguo Zhou

**Affiliations:** 1 Department of Entomology and MOA Key Lab of Pest Monitoring and Green Management, China Agricultural University, Beijing, China; 2 Department of Entomology, University of Kentucky, S-225 Agricultural Science Center North, Lexington, KY 40546-0091 USA; 3 State Key Laboratory for Biology of Plant Diseases and Insect Pests, Institute of Plant Protection, Chinese Academy of Agricultural Sciences, Beijing, China; 4 Department of Plant and Soil Sciences, KTRDC, University of Kentucky, Lexington, KY 40546 USA

**Keywords:** Reverse transcription polymerase chain reaction, Molecular biology

## Abstract

The subsocial life style and wood-feeding capability of *Cryptocercus* gives us an evolutionary key to unlock some outstanding questions in biology. With the advent of the Genomics Era, there is an unprecedented opportunity to address the evolution of eusociality and the acquisition of lignocellulases at the genetic level. However, to quantify gene expression, an appropriate normalization strategy is warranted to control for the non-specific variations among samples across different experimental conditions. To search for the internal references, 10 housekeeping genes from a gut transcriptome of a wood-feeding cockroach, *Cryptocercus punctulatus,* were selected as the candidates for the RT-qPCR analysis. The expression profiles of these candidates, including *ACT*, *EF1α*, *GAPDH*, *HSP60*, *HSP70*, *αTUB*, *UBC*, *RPS18*, *ATPase* and *GST*, were analyzed using a panel of analytical tools, including geNorm, NormFinder, BestKeeper, and comparative ΔC_T_ method. RefFinder, a comprehensive ranking system integrating all four above-mentioned algorithms, rated *ACT* as the most stable reference gene for different developmental stages and tissues. Expression analysis of the target genes, *Hex-1* and *Cell-1*, using the most or the least appropriate reference genes and a single or multiple normalizers signified this research. Our finding is the first step toward establishing a standardized RT-qPCR analysis in *Cryptocercus.*

## Introduction

### Wood-feeding *Cryptocercus*: a "missing link" between cockroaches and termites

Eusociality, in which individuals surrender their own reproduction rights to care for offspring that are not their own, is a fascinating evolutionary mystery and a complex biological trait that has intrigued scientists for decades. Tracking the evolution of this complex trait, however, is not an easy task. Studies on eusocial Hymenotpera, including bees, wasps, and ants, has been greatly facilitated by the existence of intermediates between the ancestral solitary lineages and highly evolved eusocial clades^1^. Such phylogenetic intermediates, however, are missing in Isoptera (termites are all eusocial) leading to a tremendous imbalance in sociogenomic research between Isopteran and Hymenopteran societies^2^. Multiple gene sequences analysis demonstrated that subsocial wood-feeding cockroaches in the genus *Cryptocercus*, together with termites, formed a clade nested within a larger cockroach clade, suggesting that wood-feeding cockroaches may be the best model of an evolutionary intermediate between non-eusocial cockroach taxa and eusocial termites^3^.

Besides the close phylogenetic relationship, the genus *Cryptocercus* also possesses key attributes similar to termites, including wood-feeding capability and subsocial life style with long and complex brood care^3–7^. The dual lignocellulose digestion system shared by *Cryptocercus* and termites is highly efficient. Equipped with both endogenous and symbiotic enzymes, these wood-feeding Dictyptera can convert over 90% of the recalcitrant lignocelluloses into fermentable sugars within 24 h and play a very important ecological role with respect to global forests carbon cycling and sequestration^6^. Various events have led to the separation of the ancestor group to modern *Cryptocercus,* which remains subsocial, and termites, which becomes eusocial with the evolutionary characters of division of labor, cooperative brood-care and overlapping generations^8^. *Cryptocercus*, considered a “prototermite”, is the logical and the only living intermediate, to study the evolution of eusociality in termites^9^.

### Reference gene selection: an indispensable step within the MIQE guideline

Quantitative real-time polymerase chain reaction (RT-qPCR) is, by far, the most widely used and reliable method for the detection and quantification of messenger RNA (mRNA) at the transcription level. The development of RT-qPCR leads to a sensitive, cost effective, and faster measurement of gene expression in comparison to Northern blotting, and makes the accurate quantification of gene expression over a wide concentration range reliable^10^. In addition, RT-qPCR has been adopted to validate the results from omics and functional omics analyses^11–13^. The accuracy of RT-qPCR, however, depends upon various factors, including the biological variability of samples and the technical factors associated with sample preparation, such as the quantity of starting material (e.g., cDNA concentration), RNA extraction, the integrity of RNA, storage conditions, and the efficacy of various reagents and enzymes. Therefore, normalization with internal controls (reference genes) whose expression levels are stable among different tissues, throughout all developmental stages, and/or under various treatments is critical for the accurate quantification of gene expression.

To ensure the reliability of research and integrity of scientific literature, to promote consistency and transparency among laboratories, and to streamline data analysis and interpretation, Bustin and colleagues (2009) proposed a set of MIQE (the Minimum Information for Publication of Quantitative Real-Time PCR Experiments) guidelines to the scientific community as a whole^14^. Selection of suitable reference genes is an indispensable step of the MIQE guidelines.

Historically, housekeeping genes, such as *actin* (*ACT*), *glyceraldehyde-3-phosphate dehydrogenase* (*GAPDH*), and *ribosomal RNAs* (*rRNAs*)^15^, have been used extensively as the internal references for RT-qPCR analysis without empirical validations. Under specific experimental conditions, however, their expression may vary substantially^16–18^. Consequently, there is a growing awareness to select suitable reference genes prior RT-qPCR analysis. This is especially true for non-model organisms, which are currently lagging behind well characterized model organisms in terms of genomic resources and empirically tested reference genes. As a result, researchers have started to embrace the MIQE guidelines and adopted the concept of using multiple rather than a single normalizers^19–21^. In addition, both systematic and customized studies are encouraged for each organism to identify suitable reference genes^22,23^.

### Goals and objectives

The overall goal of this study is to screen for internal references for the temporal and spatial gene quantification in a wood-feeding cockroach, *C. punctulatus.* Our overarching hypothesis is that housekeeping genes represent a rich reservoir for searching the internal references for RT-qPCR analysis. To test this hypothesis, we investigated the expression profiles of ten housekeeping genes and two target genes under the temporal and spatial conditions. The candidates included *actin* (*ACT*), *elongation factor-1α* (*EF1α*), *glyceraldehyde 3 phosphate dehydrogenase* (*GAPDH*), *heat shock protein 60* (*HSP60*), *heat shock protein 70* (*HSP70*), *α-tubulin* (*αTUB*), *ubiquitin conjugating enzyme* (*UBC*), *ribosomal protein S18* (*RPS18*), *adenosinetriphosphatase* (*ATPase*) and *glutathione-S-transferase* (*GST*) from *C. punctulatus*. Target genes, *hexamerin-1* (*Hex-1*) and *β-1,4-endoglucanase* (*Cell-1*), play a critical role in caste differentiation and cellulose degradation^24,25^, respectively, and serve as the positive controls. The temporal (developmental stage) and spatial (tissue type) expression profiles of these candidates were evaluated comprehensively by a panel of analytic programs, including geNorm, Normfinder, BestKeeper, and comparative ΔC_T_ method. Ultimately, a specific set of reference genes is recommended by RefFinder, a comprehensive ranking system integrating all four algorisms.

The advent of the next generation sequencing technologies has propelled entomological research into the Genomic Era. As the most primitive extant member of the Blattaria and the sister group of modern termites, *Cryptocercus* is the only evolutionary intermediate between cockroaches and termites. This evolutionary “missing” link represents the key species to address some major outstanding questions in biology (e.g., the evolution of eusociality). Results from this study will facilitate our efforts to (1) standardize the gene quantifications in *C. punctulatus*, (2) functionally decipher the newly sequenced and assembled *C. punctulatus* genome (unpublished data), and (3) decode the genetic basis governing the transition from solitary cockroaches to eusocial termites and the acquisition of symbiotic lignocellulolytic enzymes within woodroach-termite lineage.

## Results

### Validation of primer sets

The specificity of individual primer sets was evaluated using both gel electrophoresis and melting curve analyses. The banding pattern on 1% agarose gel showed a single band for candidate and target genes individually. Fluorescence data were collected for melting curve analysis, and a single peak was produced by each candidate as well as target gene. Linear regression coefficient for the reproducibility of RT-qPCR (R^2^) exceeded 0.99 for all the candidate reference genes and target genes, while amplification efficiency (E%) ranged between 94.1 and 109.3% , suggesting a highly specific and efficient primer design (Table S1 and Table S2).

### Optimal cDNA concentration for *GAPDH*

**Figure 1:**
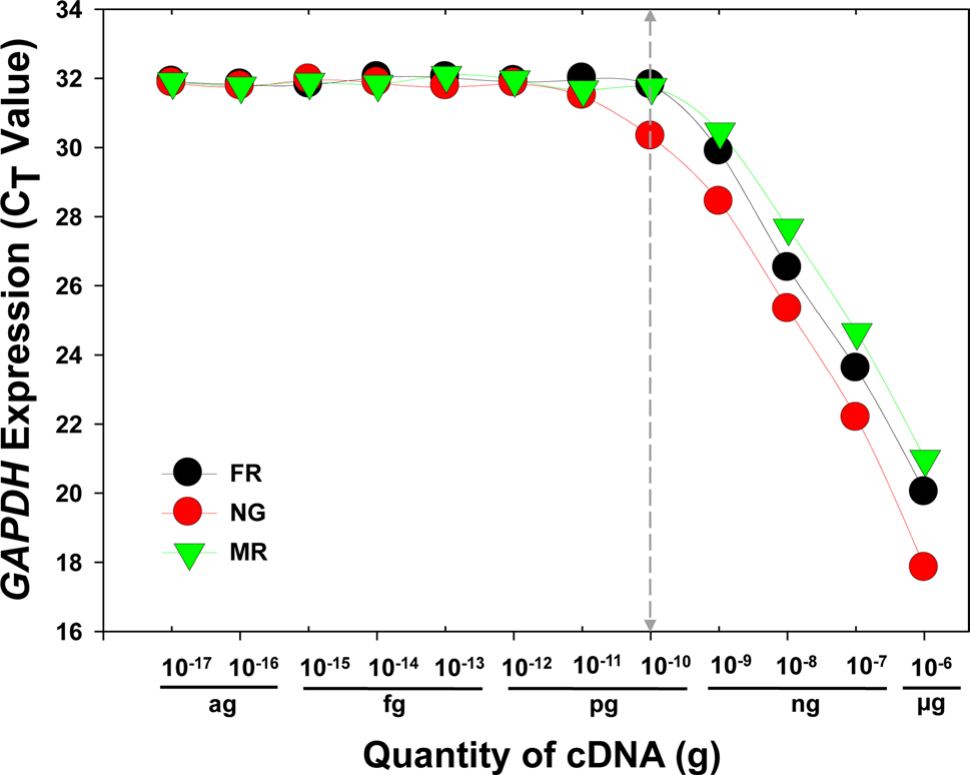
Optimal cDNA concentrations for RT-qPCR analysis. cDNAs synthesized from three different tissues FR (female reproductive organ, ovary), NG (neuron ganglion) and MR (male reproductive organ, testis) were subjected to a tenfold serial dilution before engaging in the subsequent RT-qPCR analysis.

The correlations between the C_t_ value of *GAPDH* and a gradient of cDNA concentrations generated from three different tissues were shown in Fig. 1. For reproductive organs, ovary (FR) and testis (MR), there was a positive linear relationship between C_t_ values and cDNA concentrations ranging from 0.1 ng to 1 µg. Similarly, a positive correlation was observed in neuron ganglion (NG) between C_t_ values and cDNA concentrations ranging from 0.01 ng to 1 µg (Fig. 1). Consequently, the minimum quantity of cDNAs needed for accurate quantification of *GAPDH* expression in *C. punctulatus* is approximately 0.1 ng.

### Relative gene expressions among different developmental stages and tissues

**Figure 2:**
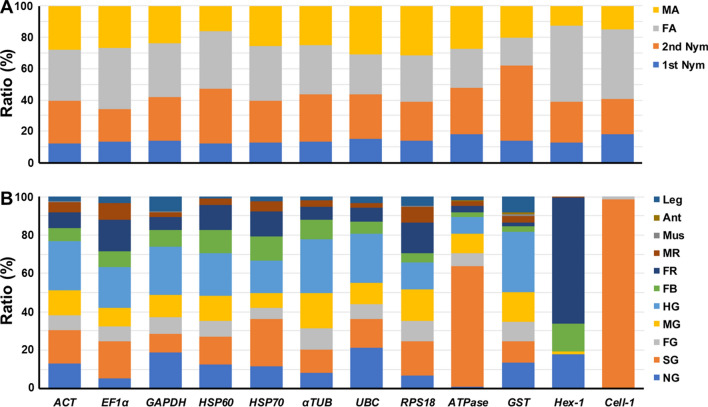
Relative expression ratios of candidate genes among different developmental stages and tissues. Relative expression ratio (%) is shown among (**A**) different developmental stages, including 1st Nym (1st nymph) and 2nd Nym (2nd nymph), MA (male adult), FA (female adult); and (**B**) different tissues, including NG (neuron ganglion), SG (salivary gland), FG (foregut), MG (midgut), HG (hindgut), FB (fatbody), FR (female reproductive organ, ovary), MR (male reproductive organ, testis), Mus (muscle), Leg, and Ant (antenna). The summation of a specific gene expression levels from samples of all the developmental stages or tissues are respectively regarded as 100%.

Throughout different developmental stages, all candidate genes exhibited the highest expression level in adult females, and the lowest expression level in the 1st nymphs (Fig. 2A; Table S3). The results from different tissues illustrated that all candidate genes showed notably different expression patterns, especially the target genes (Fig. 2B; Table S4). *Hex-1,* a negative regulator of worker-soldier caste differentiation, exhibited significantly higher expressions in the ovary (FR) and fat body (FB). *Cell-1*, a highly conserved endogenous endoglucanases, resided predominantly in the salivary gland (SG). These results demonstrated that the expression profile of housekeeping genes, although relatively stable in comparison to target genes, could vary among different developmental stages and tissues, signifying the importance and necessity for the selection of suitable reference genes.

### Stability analysis

**Figure 3:**
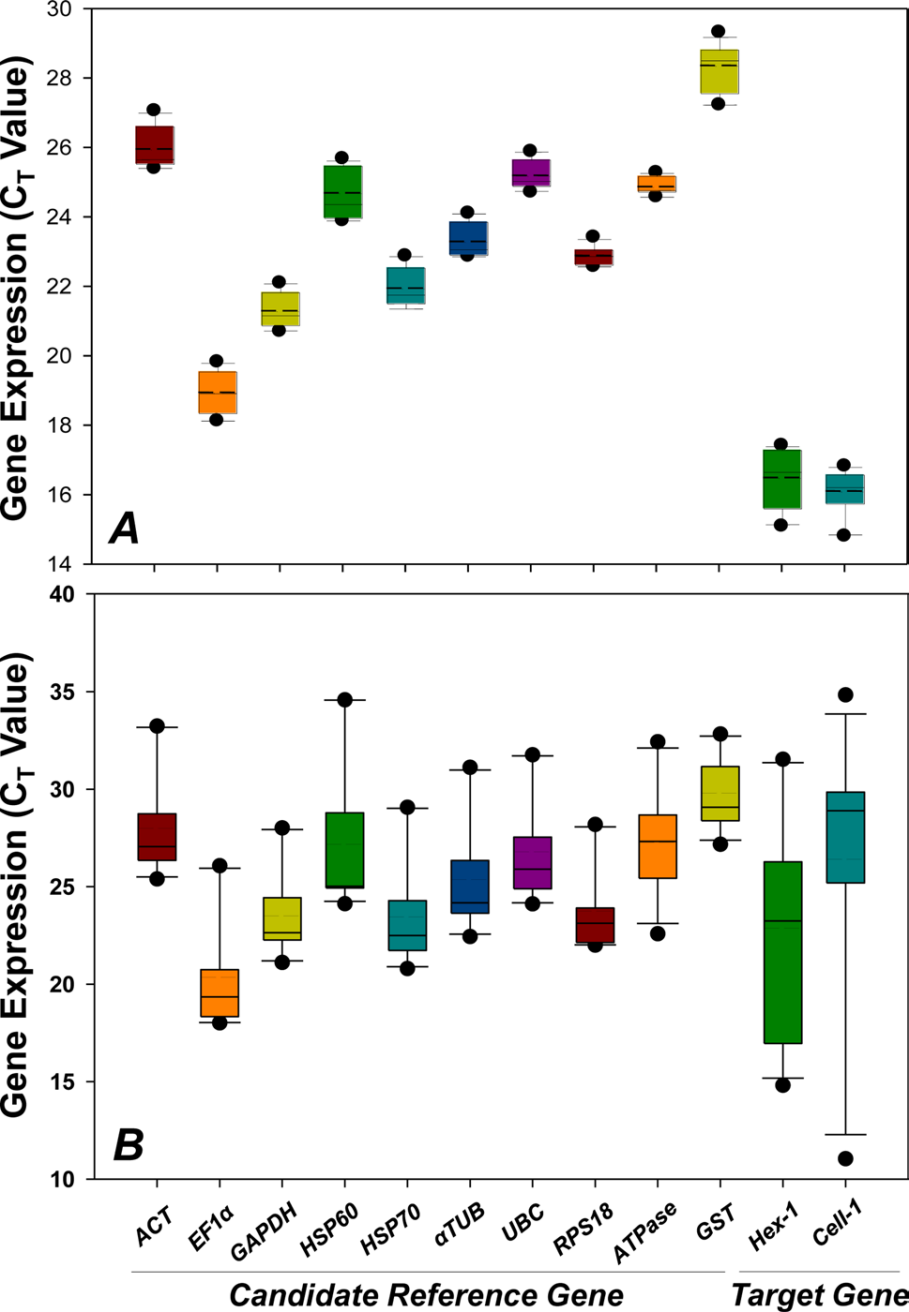
Variability of candidate genes at mRNA level among different experimental conditions. BoxPlots of (**A**) different developmental stages and (**B**) different tissues were generated from raw C_T_ values of ten candidate reference genes and two target genes examined by RT-qPCR analysis. The plots denote median, upper and lower quartiles, and 10th and 90th percentile of data. Dashed lines within bars denote the means.

Based on the C_t_ values and BoxPlot analysis (SigmaPlot 10.0), the dispersal of expressions in candidate reference genes displayed range, extreme values and outliers (Fig. 3A,B). Among them, the expression profiles of *ATPase*, *RPS18*, *UBC*, and α*TUB* were relatively stable throughout different developmental stages (Fig. 3A), whereas *RPS18*, *GAPDH*, *UBC*, *HSP70*, *ACT* and α*TUB* were relatively stable across different tissues (Fig. 3B).

geNorm calculates M-value (stability value) for each candidate reference gene and genes with a lower M-value (below the threshold value of 1.5) were considered stable. For different developmental stages, α*TUB* was the most stable candidate with the lowest M value, while *ACT* was the most stable reference gene among tissues (Table 1). BestKeeper calculates the SD and *r* value of each reference gene. Genes with a SD value < 1.0 and *r* value > 0.9 are considered stable. Candidate with the lowest SD and the highest *r* values was identified as the most stable reference gene. *GAPDH* was the most stable candidate throughout developmental stages, while *RPS18* was the one among different tissues (Table 1). NormFinder calculates gene stability through an ANOVA -based algorithm and genes showing the lowest stability values (below the threshold value of 1) are consider stable. *GAPDH* and *EF1α* were the most stable candidates for different developmental stages and tissues, respectively (Table 1). The comparative ΔC_t_ method also ranks the stability of reference gene through a stability value, in which genes with a lower stability values were considered with a higher level of stability. As a result, *ACT* and *HSP70* were the most stable candidates throughout developmental stages, while *ACT* was also the most stable reference gene among tissues (Table 1).

**Table 1:** Ranking of candidate reference genes.

geNorm Ranking	Developmental stage		Tissue			
	Gene name	M-value	Gene name	M-value		
1	*αTUB*	0.300	*ACT*	0.892		
2	*GAPDH*	0.307	*UBC*	0.991		
3	*HSP70*	0.316	*αTUB*	1.018		
4	*ACT*	0.329	*HSP70*	1.038		
5	*UBC*	0.371	*EF1α*	1.043		
6	*RPS18*	0.387	*RPS18*	1.123		
7	*ATPase*	0.408	*GAPDH*	1.144		
8	*EF1α*	0.453	*ATPase*	**1.599**		
9	*HSP60*	0.551	*HSP60*	**1.630**		
10	*GST*	0.717	*GST*	**1.709**		

BestKeeper Ranking	Developmental stage			Tissue		
	Gene name	SD	[*r*]	Gene name	SD	[*r*]
1	*GAPDH*	0.37	0.977	*RPS18*	0.56	0.975
2	*αTUB*	0.40	0.984	*GAPDH*	0.74	0.959
3	*ACT*	0.43	0.999	*ATPase*	0.91	0.889
4	*HSP70*	0.45	0.996	*UBC*	0.96	0.981
5	*UBC*	0.31	0.893	*ACT*	0.99	0.995
6	*EF1α*	0.47	0.898	*EF1α*	**1.03**	0.982
7	*RPS18*	**1.24**	0.977	*HSP70*	**1.14**	0.984
8	*ATPase*	0.51	**0.043**	*αTUB*	**1.19**	0.988
9	*GST*	0.59	**0.598**	*HSP60*	**1.98**	0.987
10	*HSP60*	0.66	**0.772**	*GST*	**1.52**	**0.837**

Normfinder Ranking	Developmental stage		Tissue			
	Gene name	Stability Value	Gene name	Stability Value		
1	*GAPDH*	0.036	*EF1α*	0.145		
2	*αTUB*	0.038	*ACT*	0.166		
3	*HSP70*	0.088	*HSP70*	0.190		
4	*ACT*	0.145	*UBC*	0.206		
5	*UBC*	0.230	*αTUB*	0.212		
6	*RPS18*	0.231	*RPS18*	0.492		
7	*EF1α*	0.236	*GAPDH*	0.598		
8	*ATPase*	0.238	*ATPase*	0.813		
9	*HSP60*	0.258	*HSP60*	0.816		
10	*GST*	0.491	*GST*	**1.235**		

Comparative ΔCt Ranking	Developmental stage		Tissue			
	Gene name	Stability Value	Gene name	Stability Value		
1	*ACT*	0.46	*ACT*	0.90		
2	*HSP70*	0.46	*UBC*	1.00		
3	*αTUB*	0.46	*αTUB*	1.02		
4	*GAPDH*	0.47	*HSP70*	1.04		
5	*UBC*	0.57	*EF1α*	1.04		
6	*EF1α*	0.57	*RPS18*	1.13		
7	*HSP60*	0.70	*GAPDH*	1.15		
8	*GST*	0.87	*ATPase*	1.60		
9	*ATPase*	1.08	*HSP60*	1.67		
10	*RPS18*	1.26	*GST*	1.71		

The bold indicates the gene expression is unstable under specific experimental conditions.

**Figure 4:**
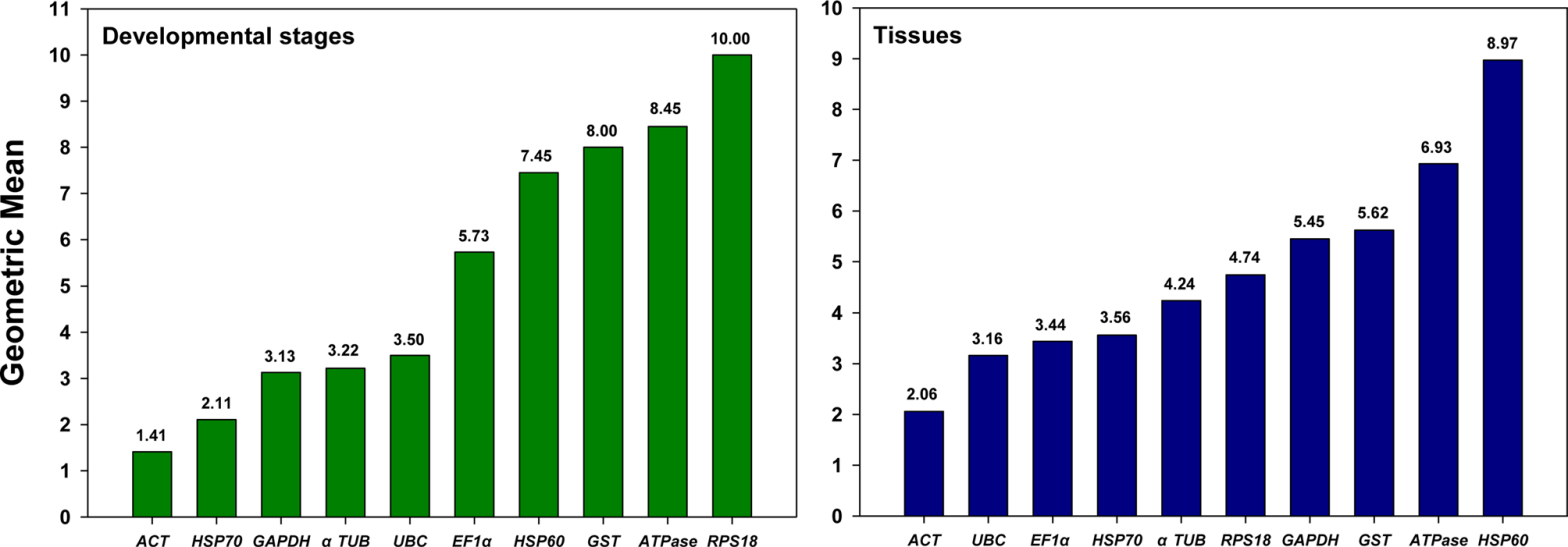
Expression stability of candidate reference genes. The geometric means of the expressional stability were comprehensively calculated by RefFinder for candidate reference genes in different developmental stages and tissues, and the lower value of geometric mean denotes higher expressional stability.

Finally, RefFinder provides the most comprehensive ranking by integrating the geomean of stability values derived from all four analytic tools. For developmental stages, the rank of candidates from the most to the least stable was *ACT* > *HSP70* > *GAPDH* > *αTUB* > *UBC* > *EF1α* > *HSP60* > *GST* > *ATPase* > *RPS18,* while, for different tissues, it was *ACT* > *UBC* > *EF1α* > *HSP70* > *αTUB* > *RPS18* > *GAPDH* > *GST* > *ATPase* > *HSP60* (Fig. 4).

### The optimal number of reference genes

**Figure 5:**
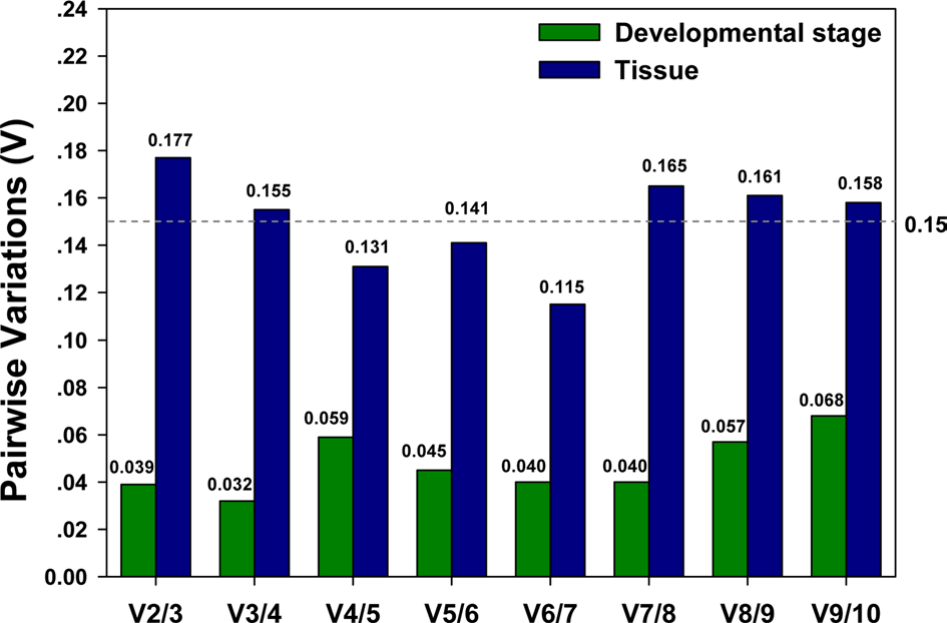
Pairwise variation analysis by geNorm. Optimal number of reference genes required for accurate normalization of target transcript expressions among different developmental stages and different tissues were determined by geNorm analysis based on pairwise variations of *V*_*n/n*+*1*_.

To search for the optimal number of reference genes, geNorm calculates all pairwise variations under each experimental condition (Fig. 5). Based on Vandesompele and colleagues^26^, a Vn/Vn + 1 threshold value of 0.15 suggests that the addition of “N + 1” reference gene is not necessary, i.e., “N” number of references genes is sufficient to normalize qRT-PCR results. For developmental stages, *V*_*2/3*_ was lower than 0.15, indicating that *ACT* and *HSP70* were sufficient for the accurate normalization (Fig. 5). For tissues, however, the first *V* value less than the threshold was at *V*_*4/5*_, suggesting that *ACT*, *UBC*, *EF1α* and *HSP70* were the best combination for the precise normalization (Fig. 5).

### Validation of selected reference genes with target genes *Hex-1* and *Cell-1*

**Figure 6:**
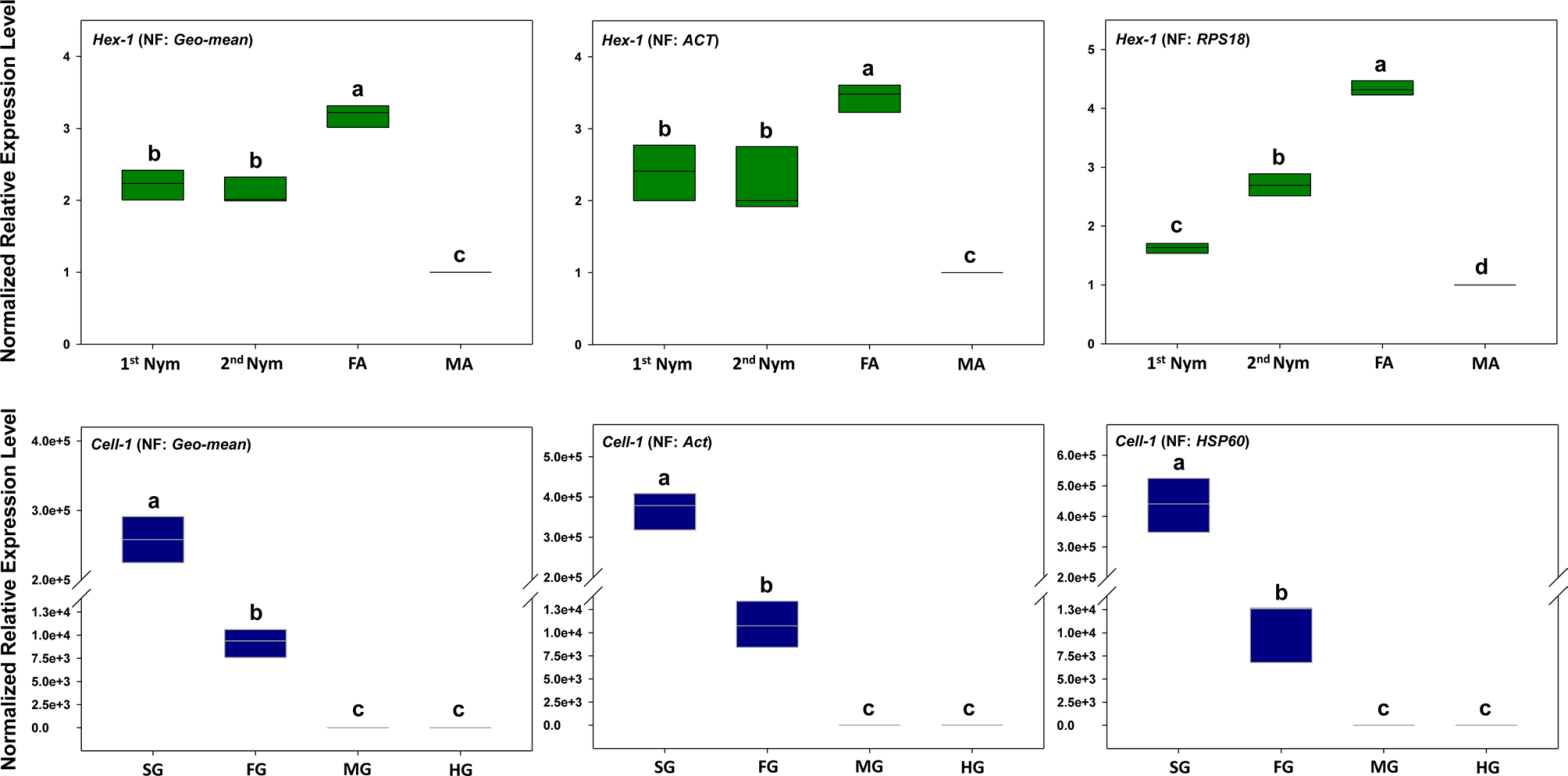
Comparative RT-qPCR analysis of target gene expressions based on different reference genes. Among different developmental stages (**UPPER**), transcriptional profiles of target genes *Hex-1* were determined with the recommended multi-gene normalizer (*ACT* + *HSP70*), the single best endogenous reference gene *ACT* and the single worst endogenous reference gene *RPS18*, respectively. Among different tissues (**LOWER**), expression patterns of *Cell-1* were determined with the recommended multi-gene normalizer (*ACT* + *UBC* + *EF1α* + *HSP70*), the single best endogenous reference gene *ACT* and the worst endogenous reference gene *HSP60*, respectively. Different letters denote significant expression differences among the three normalizers using one-way ANOVA test (*p* < 0.05) by SPSS (IBM SPSS Statistics 20).

The expression profiles of *Hex-1* and *Cell-1,* the target genes, were evaluated to validate the recommended reference genes under different biotic conditions. Across different developmental stages, the expression profile of *Hex-1* was similar when normalized to the most stable reference gene *ACT* and the recommended multi-gene normalizer (*ACT* and *HSP70*). The expression of *Hex-1* was significantly different when it was normalized to the least stable reference gene *RPS18* (Fig. 6). Specifically, the expression of *Hex-1* was significantly underestimated in the 1st nymphs.

Among different tissues, similar expression profiles of *Cell-1* were observed when *Cell-1* was normalized to the most stable reference gene *ACT*, the recommended multi-gene normalizer (*ACT*, *UBC*, *EF1α* and *HSP70*), and the least stable gene *HSP60*. Although the expression profiles were similar, *Cell-1* expressions in both salivary gland and foregut were overestimated, especially when *HSP60* was used as the normalizer (Fig. 6).

## Discussion

### Selection of candidate reference genes

It is unrealistic to find a “universal” normalizer showing constant expression level across all experimental conditions. In this study, expressions of candidate reference genes varied, more or less, among different developmental stages and tissues. Changes in C_t_ values ≥ 1.0 represent ≥ twofold changes in gene expression level, i.e., small variability in C_t_ values could have drastic impact on target gene expression^27^. Consequently, selection and validation of genes exhibiting a relative low variability under specific experimental conditions is a critical step toward accurate gene quantification study.

A suitable reference gene should have consistent transcription in all types of cell/tissue types at specific testing conditions, and the transcription of such gene should not be regulated by either internal or external factors^28^. Additionally, the expression level (C_t_ value) of target and reference genes should be comparable to ensure that all transcripts are subject to the same kinetic interactions during qRT-PCR^26^. Otherwise, the expression of a highly abundant internal reference (e.g., ribosomal proteins with significant lower C_t_ values) can mask the subtle, but potentially biologically relevant, changes in the expression of target genes^29^. Although the number of reference gene selection publications has been steadily increased for the past decade, the average number of reference genes been tested was 9.53^15^. In this study, we selected ten housekeeping genes, which have a track record of being used as the internal controls, as the reference gene candidates. Target genes, *hexamerin-1* (*Hex-1*) and *β-1,4-endoglucanase* (*Cell-1*) are of primary importance for caste differentiation and cellulose degradation research. The expression levels of target and candidate reference genes were comparable, with C_t_ values ranging between 16 and 25 using cDNAs generated from the whole body of *C. punctulatus* adults.

Previous studies have demonstrated the significant impacts of tissue/cell types and developmental stages on the stability of reference gene expression, in some case, even greater than treatments^30–33^. Here, we empirically examined the temporal and spatial stability of these candidate genes, and recommended different sets of reference genes for tissue/cell types and developmental stages, respectively.

### Stability assessment

Although the underlying algorithms employed by each analytical tool are different, they all focus on the variance in C_t_ values of each reference gene across treatments^34^. In this study, reference genes recommended by the four analytical tools exhibit some discrepancies, albeit share some commonalities. For different developmental stages, *GAPDH* was rated as the most stable reference gene by both BestKeeper and Normfinder, whereas α*TUB* and *ACT* were the top choice by geNorm and comparative ΔC_t_ method. Similarly, *GAPDH* was the reference gene of choice in a few lepidopterans, including the silkworm *Bombyx mori*, *Chilo suppressalis*, the pink stem borer *Sesamia inferens,* and the oriental leafworm moth *Spodoptera litura*^35–38^, and optimal reference gene for profiling of seasonal and labor-specific gene in Western honey bee, *Apis mellifera*^16^. *ACT* was also considered the most stable reference gene in the western corn rootworm, *Diabrotica virgifera virgifera*, the striped rice stem borer, *C. suppressalis* and the Jackfuit borer, *Diaphania caesalis*^35,36,39^. However, the least stably expressed candidate in *C. punctulatus*, *RPS18,* showed the highest stability in the pink spotted lady beetle, *Coleomegilla maculate*, the housefly, *Musca domestica* and *A. mellifera* ^16,40,41^.

For tissues, both geNorm and comparative ΔC_t_ method ranked *ACT* as the most stable reference gene, while *RPS18* and *EF1α* were, respectively, recommended by BestKeeper and Normfinder. Robledo and colleagues^34^ used a set of empirical data evaluated the accuracy of BestKeeper, Normfinder, geNorm, and comparative ΔC_t_ method. Authors suggested that NormFinder, complemented with the descriptive statistics calculated by BestKeeper, offers the most reliable recommendation. In this study, NormFinder selected *GAPDH* and *EF1α* as the most stable reference genes, respectively, for developmental stages and tissues (Table 1). Indeed, *EF1α* has been picked as the most stable reference genes across different tissues in many insects, such as bed bug, *Cimex lectularius*, bumble bee, *Bombus lucorum*, diamondback moth, *Plutella xylostella* and oriental armyworm, *Mythimna separata*^19,42–44^.

The commonality and discrepancies displayed here confirm the notion that no universal reference genes exist for all contexts and reference gene selection and validation is crucial for accurate quantification of gene expression under specific experimental conditions. Without these studies, single un-validated endogenous controls can have profound impacts on data analysis and lead to questionable interpretation^16,18,19,45,46^. In this study, the expression of *Hex-1* was significantly underestimated in the 1st nymphs when the least stable instead of the most stable and recommended reference genes was used to normalize target gene expression. Similarly, *Cell-1* expressions in both salivary gland and foregut were overestimated when we elected the least stable instead of the most stable and recommended reference genes (Fig. 6). This is consistent with other validation studies that compared the use of stable *vs* unstable reference genes in the estimation of the target gene expression, in which normalization to unstable reference genes led to over- or under-estimated expressions in the target genes^47–49^.

### Optimal number of reference genes: single *vs* multiple normalizers

Besides stability, the number of reference genes used for normalization in a specific experiment can impact RT-qPCR analysis as well. Suzuki and colleagues reported that over 90% of the RNA transcription analysis published in peer-reviewed journals used a single housekeeping gene as reference^50^. Housekeeping genes, such as *GAPDH*, *ACT*, and *RPS18*, have been used extensively as the single reference gene without empirical validation, however, many of these reference genes showed substantial variations at expression level under different experimental conditions^17,51–53^. In fact, as the pool expanded, the chance of these “generic” candidates to be the reference gene of choice decreases^34^. Since the introduction of MIQE guidelines in 2009, researchers have grown more receptive to adopt multiple rather than a single reference gene in RT-qPCR analysis. Despite changes in perception, the implementation of these guidelines has been challenging. The average number of reference genes used in peer-reviewed publications between 2010 and 2015 remained 1.23, in which 13% of the studies used more than a single reference gene^34^.

The optimal number of reference genes in a specific study is suggested by geNorm based on the calculation of normalization factors (NFs) in parallel samples. Pairwise variation (*V*_*n/n*+*1*_) is obtained from NF ratios between N and N + 1 reference genes. The minimum *V*_*n/n*+*1*_ on a U-shape curve composed by all the *V*_*n/n*+*1*_ represents the most stable NF that can be obtained among all the reference genes in a specific sample set. The number “N” corresponds to the optimal number of reference genes that are needed for the most accurate data normalization^26^. In this study, geNorm showed that all the *V* values were below the threshold among different developmental stages, with *V*_*3/4*_ had the lowest pairwise variation value of 0.032. However, we elected to recommend two reference genes instead of three as the optimal number because *V*_*2/3*_ value of 0.039 was equally low and far more practical and economical. Similarly, although *V*_*6/7*_ (0.115) predicted the best number of reference genes for different tissues, four was the number of choice for the same set of reasons (*V*_*4/5*_ = 0.131; Fig. 5).

Interestingly, it seems that more samples involved in the experiment (4 developmental stages *vs* 11 tissues) demand a higher number of reference genes (2 *vs* 4) for accurate normalization. A plausible explanation for this phenomenon is that when more samples were added into the analysis, *V*_*n/n*+*1*_ would be slower to reach the minimum value due to the introduction of more unstable factors. Consequently, there is no fixed number of internal controls for gene expression studies. The optimal number of reference genes for accurate normalization can be influenced by *V*_*n/n*+*1*,_ sample size, and practicality/feasibility.

### cDNA concentration

The other factor which can impact the accuracy of RT-qPCR analysis is the initial concentration of cDNA template. In RT-qPCR, fluorescence is positively correlated with the amount of amplified product, suggesting the C_t_ value is cDNA concentration-dependent. In this study, the optimal range of cDNA concentration to precisely quantify *GAPDH* expression was between 0.1 ng and 1 µg for reproductive and neuron tissues. When cDNA was less than 0.1 ng, the expression of tested genes (C_t_ value) did not correlate with the quantity of cDNA template, which meant no changes could be detected. Although 0.1 ng–1 µg is specifically for *GAPDH*, accurate quantification of gene expression depends on the optimal range of cDNA concentration, i.e., the quality and quantity of cDNA template can directly impact the accuracy of RT-qPCR analysis.

## Materials and methods

### Ethics statement

Woodroaches were collected from rotting logs on the grounds of Mountain Lake Biological Station, Giles Co., Virginia (latitude 37.364, longitude 80.519). No specific permits were required for the described field studies.

### Colony maintenance

The collected woodroaches were maintained at the University of Kentucky in a ten-gallon glass aquarium under complete darkness and provisioned with brown rotted pine at 20 ± 1 °C with limited humidity. The identity of *Cryptocercus* species was determined by a combination of morphological traits and a molecular marker, 12S rRNA. Based on the diagnostic nucleic acid sites embedded in the amplified 12S rRNA fragments, collected *Cryptocercus* were identified as *C. punctulatus*^54^.

### Sample preparation

*Cryptocercus punctulatus* colonies were acclimated in the laboratory for two weeks before they were subjected to the sample preparation. *Cryptocercus punctulatus* colony typically contains a pair of reproductives (adult male and female) and different-sized nymphs.

For developmental stages, we collected four 1st nymphs (1st Nym), three 2nd nymphs (2nd Nym) and one adult male (MA) and one adult female (FA) to represent respective developmental stages within a colony. A total of three colonies were used in this experiment, and each colony represented a biological replication.

For different tissues, leg (Leg), antenna (Ant), muscle (Mus), neuron ganglion (NG), salivary gland (SG), foregut (FG), midgut (MG), hindgut (HG), fatbody (FB), ovary (FR), and testis (MR) were individually dissected from *C. punctulatus* adults. Before dissection, *C. punctulatus* were surface sterilized in 70% ethanol for 1 min and followed by rinsing in sterile water for 30 s. *Cryptocercus punctulatus* adults were dissected under a binocular microscope in 10 mM phosphate buffered saline (PBS, pH 7.8), and respective tissues were snap frozen in liquid nitrogen and stored at -80 °C. Dissected individual tissue samples from three same-sex adults were pooled to represent one tissue type in one biological replication. A total of three biological replications were carried out for this experiment.

### Total RNA extraction and cDNA synthesis

*Cryptocercus punctulatus* whole body or dissected tissues was snap frozen in liquid nitrogen, and then ground to powder using a mortar and pestle. To preserve the integrity of RNA, the grinding process was carried out in liquid nitrogen. The resultant ground up powder (≤ 30 mg) was transferred to a 1.5 ml microcentrifuge tube for RNA extraction using a SV Total RNA Isolation Kit (Promega, Madison, WI, USA) according to the manufacturer’s instruction. DNA contamination was eliminated by the DNAase treatment for 15 min. Quality and quantity of total RNA was measured using a NanoDrop 2000 spectrophotometer (Thermo Fisher, USA). cDNA was synthesized using the resultant total RNA as the template and M-MLV transcriptase (Grand Island, NY, USA). Samples without reverse transcriptase were used as the negative controls to make sure there was no contamination of DNA.

### Selection of candidate reference genes and design of RT-qPCR primers

The selection of candidate reference genes in this study has followed three criteria: (1) they must be housekeeping genes, which are constitutively expressed in all cells/tissue types and maintain basic cellular functions; (2) they have been used historically/extensively as internal references for gene quantification studies in other organisms; and (3) they are presented in a *C. punctulatus* transcriptome (unpublished data). Based on these criteria, we selected ten housekeeping genes, *actin* (*ACT*), *elongation factor-1α* (*EF1α*), *glyceraldehyde 3 phosphate dehydrogenase* (*GAPDH*), *heat shock protein 60* (*HSP60*), *heat shock protein 70* (*HSP70*), *α-tubulin* (*αTUB*), *ubiquitin conjugating enzyme* (*UBC*), *ribosomal protein S18* (*RPS18*), *adenosinetriphosphatase* (*ATPase*) and *glutathione-S-transferase* (*GST*), as the candidates with accession numbers from JQ686945 to JQ686954, respectively. Target genes, *hexamerin-1* (*Hex-1*) and *β-1,4-endoglucanase* (*Cell-1*), were extracted from the same transcriptome (unpublished data) with accession numbers JQ686955 and JQ686956, respectively.

Primers were designed by Primer3 (SimGene.com) (Supplementary Table S2), synthesized and diluted to a working concentration of 10 µM. RT-qPCR reactions were run in triplicate on a Bio-Rad MyiQ™ Single-Color Real-Time PCR Detection System (BioRad, Hercules, CA). The thermal cycling profile included an initial denaturation step at 95 °C for 5 min, followed by 40 cycles of 95 °C for 15 s, annealing at 53 °C for 45 s, and concluded by an extension step at 72 °C for 30 s. Samples were run on 1% agarose gel, and then run with the dissociation protocol for melting curve analysis to check the specificity of each individual primer sets. In addition, amplification efficiency (E%) and correlation coefficient (R^2^) were determined based on the standard curves generated from a tenfold serial dilution of cDNAs.

### Optimal cDNA concentration for RT-qPCR analysis

cDNAs from ovary (FR), neuron ganglion (NG) and testis (MR), respectively, were quantified using a Smart Spec Plus spectrophotometer (Bio-Rad, Hercules, CA). A tenfold serial dilution was carried out to generate a cDNA concentration gradient ranging from 10^–6^ to 10^–17^ g. After RT-qPCR, C_t_ (Threshold Cycle, which is the number of cycles required for the fluorescent signal to exceed the threshold line of background level) values of *GAPDH* transcripts corresponding to a gradient of cDNA concentrations were analyzed, and the optimal range of cDNA concentrations was determined.

### Stability analysis

Relative expression level of the ten candidate reference genes and the two target genes were calculated by 2^−ΔCt^ method^55^. The relative expression levels of candidate reference genes across different developmental stages and tissues were analyzed using one-way ANOVA with SPSS Statistics 17.0 (SPSS Inc., Chicago, IL, USA). The means were compared by Tukey test, if the data fit homoscendasticity, and Games-Howell test were performed if not. Specifically, throughout different developmental stages, Tukey test was used for *EF1α*, *GAPDH*, *HSP70*, *αTUB*, *UBC*, *GST* and *Hex-1*, while Games-Howell test was carried out for *ACT*, *HSP60*, *RPS18*, *ATPase* and *Cell-1*. Relative expression of all the candidate reference genes across different tissues was analyzed using Games-Howell test. The dispersion of C_t_ values was assessed using a Box Plot.

The expression profiles of the candidate reference genes and target genes under different biotic conditions (developmental stages and tissues) were evaluated individually using a panel of analytic tools, including geNorm^26^, BestKeeper^56^, Normfinder^57^ and the comparative ΔC_t_ method^58^. For geNorm, each reference gene is evaluated by calculating the pairwise variation with all other genes to determine the gene-stability value, M^26^. BestKeeper ranks the reference genes based on the standard deviation (SD) of C_t_ value and the repeated pairwise correlation analyses of all the candidate genes^56^. Instead of measuring the overall stability, Normfinder selects reference genes based on the possible intra- and inter- group variation across different samples^57^. The comparative ΔC_t_ method ranks the reference genes by comparing relative expression of “pairs of genes” within each sample, and the stability of the candidates was obtained according to the repeatability of the gene expression differences among different samples^58^. The final composite ranking of stability, however, was provided by RefFinder^59^ (http://150.216.56.64/referencegene.php). RefFinder, a web-based analysis tool, assigns an appropriate weight of the four above mentioned analytical tools to an individual gene and calculates the geometric mean of their weights for the overall ranking.

Relative expression of the target genes, *Hex-1* and *Cell-1*, was calculated using ΔΔCt method^60^. Differences in their expression using an array of normalization factors were compared according to one-way ANOVA with Tukey test.

## Supplementary Information

Supplementary Information.
