# Supplementary material for: Reference gene selection for transcriptional profiling in Cryptocercus punctulatus, an evolutionary link between Isoptera and Blattodea
Source: Sci Rep. 2020 Dec 17;10:22169. doi: 10.1038/s41598-020-79030-6 (PMC7746730; doi:10.1038/s41598-020-79030-6)
Supplement: Supplementary file 1 — Supplementary Information. [file 41598_2020_79030_MOESM1_ESM.pdf]

Supplementary Materials

Reference gene selection for transcriptional profiling in *Cryptocercus punctulatus*, an evolutionary link between Isoptera and Blattodea

Zhen Li[1,2,#], Xiangrui Li[2,3,#], Qingwen Zhang[1], Ling Yuan[4], Xuguo Zhou[2,*]

[1] *Department of Entomology and MOA Key Lab of Pest Monitoring and Green Management, China Agricultural University, Beijing, China*

[2] *Department of Entomology, University of Kentucky, Lexington, KY 40546-0091, USA*

[3] *State Key Laboratory for Biology of Plant Diseases and Insect Pests, Institute of Plant Protection, Chinese Academy of Agricultural Sciences, Beijing, China*

[4] *Department of Plant and Soil Sciences, KTRDC, University of Kentucky, KY 40546, USA*

[#] These authors contribute equally.

----

* Corresponding Author:

Dr. Xuguo "Joe" Zhou

Department of Entomology

University of Kentucky

S-225 Agricultural Science Center North

Lexington, KY 40546-0091

Phone: 859-257-3125

Fax: 859-323-1120

Email: xuguozhou@uky.edu

Table S1 Primers used for RT-qPCR analysis

Gene	Forward primer	Reverse primer	Amplicon (bp)	R^{2*}	E (%)§
Candidate reference gene					
Actin	AACAAGGAGCCTCAATTCATCATC	ACCAGTAGCATCAAAAGCCTCATC	144	0.9973	105.2
EF1α	CGGTCGCATTTCTCCTTGATTTC	TGGTTATGTGGCTGGCGACTC	180	0.9997	98.7
GADPH	GCGCAGCCCAGAACATTATCC	GCCGACTTGCCCAACCTGAC	158	0.9997	101.2
HSP60	GCTCAACGGAAACCATTAGTCATC	AAAACCAGGGGCCTTCACAG	122	0.9998	97.2
HSP70	GCGTTCTCCATGACCCTTCTTATC	TTCCTGCCTACTTCAACGATTCC	146	0.9997	99.0
αTUB	TGGGGATGTAGTACCGAAAGATG	GTAGGGGGCTGGTAGTTGATGC	124	0.9998	97.9
UBC	TCCTGCGGTTCAATGAGTAGTATC	CAGTTTTCCAATTGCCAGGTG	131	0.9972	94.7
RPS18	TGCATATCGGCGACCAACTC	GTGCTGATCTGCTAAAGGAAAATG	144	0.9998	94.1
ATPase	CCAGCCCGGAAGACAAAAC	GCCAGCCACAGGTAGAAGATG	135	0.9994	94.3
GST	GCCAGGCAGGTAGTAGAAATCAATAG	TACGGGTGGAAGTTGTCAGAAG	122	0.9902	109.3
Target gene					
Hex-1	ACGTGGCTCCTGTCAATGTAG	CGCAAATCCCACGACTCC	121	0.9998	98.9
Cell-1	GGGATTGGCGCTGTTGTAG	CACCCACTCATCCGCATC	94	0.9996	96.6

*: Linear regression coefficient for the reproducibility of qRT-PCR analysis.

§: Amplification efficiency calculated by the equation $E=10^{(-1/slope)}$.

Table S2 Functions of candidate reference and target genes

Gene	Full Name	Function
Candidate reference gene		
Actin	*actin*	cytoskeletal structural protein
EF1α	*elongation factor-1α*	catalysation of GTP-dependent binding of amynoayl-total RNA to the ribosome; translational factor
GAPDH	*glyceraldehyde 3 phosphate dehydrogenase*	oxidoreductase in glycolysis and gluconeogenesis
HSP60	*heat shock protein 60*	preventing damage to proteins in response to high levels of heat; chaperonin to assist folding linear amino acid chains into their respective three-dimensional structure
HSP70	*heat shock protein 70*	preventing damage to proteins in response to high levels of heat
αTUB	*α-tubulin*	cytoskeletal structural protein
UBC	*ubiquitin conjugating enzyme*	protein degradation
RPS18	*ribosomal protein S18*	component of the 40S ribosome
ATPase	*ATPase*	catalyze the decomposition of adenosine triphosphate (ATP) into adenosine diphosphate (ADP)
GST	*glutathione-S-transferase*	antioxidative enzyme
Target gene		
Hex-1	*hexamerin 1*	Multifunctional insect storage proteins utilized during metamorphosis
Cell-1	*cellulase 1*	catalyze cellulolysis

Table S3. Expression profiles of candidate and target genes across developmental stages

Gene	1st Nymph*	2nd Nymph	Female	Male
Candidate reference gene				
ACT	1.11±0.06 b	2.53±0.290 a	2.99±0.096 a	2.59±0.047 a
EF1α	1.05±0.290 a	1.67±0.024 c	3.13±0.076 a	2.14±0.058 b
GAPDH	1.02±0.017 d	2.04±0.035 b	2.50±0.047 a	1.77±0.025 c
HSP60	1.10±0.051 b	3.08±0.131 a	3.28±0.119 a	1.46±0.308 b
HSP70	1.03±0.02 c	2.19±0.051 b	2.80±0.051 a	2.10±0.044 c
αTUB	1.01±0.008 c	2.23±0.058 a	2.30±0.042 a	1.86±0.041 b
UbC	1.02±0.017 c	1.91±0.057 ab	1.70±0.052 b	2.08±0.096 a
RPS18	4.97±2.008 b	8.76±0.301 ab	10.22±0.515 a	11.14±0.30 a
ATPase	1.03±0.020 b	1.65±0.198 a	1.41±0.012 ab	1.53±0.067 a
GST	1.25±0.124 c	4.17±0.044 a	1.58±0.084 bc	1.77±0.027 b
Target gene				
Hex-1	1.13±0.078 c	2.36±0.10 b	4.43±0.319 a	1.11±0.067 c
Cell-1	1.32±0.090 a	1.67±0.094 a	3.27±0.553 b	1.09±0.067 a

"*": Developmental stages included the 1st nymph, 2nd nymph, adult female and male. Different letters following the relative expression level denote significance.

Table S4. Expression profiles of candidate and target genes across various tissues

Gene	NG*	SG	FG	MG	HG	FB	FR	MR	Mus	Ant	Leg
Candidate reference gene											
ACT	144.23±11.080 bc	191.04±5.130 b	89.41±10.513 cd	145.08±22.190 bc	280.79±26.603 a	78.75±3.329 de	91.30±5.812 cd	58.63±5.572 def	1.47±0.154 f	1.24±0.188 f	27.37±0.714 e
EF1α	80.13±6.740 bc	280.71±12.480 a	115.07±9.681 b	143.67±22.704 bc	308.08±25.744 a	121.54±9.534 b	245.06±16.675 a	124.72±11.386 b	1.15±0.099 d	1.80±0.163 d	46.34±3.898 cd
GAPDH	95.40±6.072 b	50.22±0.691 c	45.10±2.615 cd	59.14±9.854	127.87±7.520 a	44.08±1.713 c	34.44±2.192 cd	12.80±0.140 de	1.38±0.112 e	1.07±0.039 e	39.39±1.809 c
HSP60	1267.28±107.151 bc	1462.75±40.962 b	831.93±72.041 cd	1305.79±208.370	2270.53±201.692 a	1212.96±65.663 bc	1318.98±214.021 bc	364.43±33.137 de	1.70±0.348 e	1.64±0.149 e	89.15±2.863 e
HSP70	192.99±38.306 c	411.23±21.297 a	96.958±6.306 d	129.43±20.775 cd	288.37±32.871 b	210.58±6.918 bc	215.81±15.223 bc	88.32±7.70 de	1.35±0.181 e	1.59±0.155 e	36.61±2.542 de
αTUB	130.83±7.521 cd	189.38±6.669 bc	173.01±13.227 c	289.59±54.760 b	439.86±42.662 a	164.31±8.200 c	105.55±8.077 cde	50.81±7.103 de	1.06±0.044 e	1.71±0.175 e	29.11±1.426 de
UbC	171.83±13.191 a	120.01±1.947 b	61.71±4.501 cd	89.36±11.806 bc	208.10±20.818 a	47.64±4.001 cd	59.87±3.030 cd	19.35±1.663 de	1.03±0.044 e	1.26±0.156 e	25.38±2.016 de
RPS18	33.11±1.446 d	83.33±1.866 a	50.86±4.486 bcd	79.56±13.884 ab	65.59±7.164 abc	22.47±1.455 de	76.74±9.778 ab	38.91±5.453 cd	1.14±0.087 e	1.71±0.190 e	22.25±1.686 de
ATPase	14.56±2.015 e	979.26±60.435 a	98.39±11.875 bcde	157.90±21.347 b	136.64±15.402 bc	36.57±2.415 de	56.27±1.487 bcd	36.02±3.077 cd	1.06±0.032 e	3.16±0.439 e	33.16±2.498 e
GST	28.72±0.519 bc	23.33±0.593 bc	21.00±1.970 c	32.63±5.587 b	66.65±4.020 a	5.86±0.449 e	4.18±0.213 e	7.35±0.76 de	1.34±0.192 e	1.89±0.036 e	17.68±0.435 cd
Target gene											
Hex-1	38758.52±4135.625 b	419.63±60.156 c	51.24±6.699 c	2256.67±216.799 c	108.81±9.899 c	32167.95±2174.610 b	142707.36±9115.863 a	849.45±127.910 c	1.34±0.188 c	2.38±0.190 c	384.67±20.807 c
Cell-1	196.57±27.099 b	15891390.95±1391797.460 a	213668.99±18594.514 b	32.21±3.414 b	189.25±13.872	871.69±48.120 b	66.47±2.014 b	34.69±3.363 b	1.09±0.058 b	42.27±1.437 b	57.42±2.814 b

"*": Tissues were dissected from *C. punctulatus* adults, and they included leg (Leg), antenna (Ant), muscle (Mus), neuron ganglion (NG), salivary gland (SG), foregut (FG), midgut (MG), hindgut (HG), fatbody (FB), ovary (FR), and testis (MR). Different letters following the relative expression level denote significance.
